# Reply to Özdemir, Ö. Allergic Disease with Selective IgA Deficiency. Comment on “Cinicola et al. The Allergic Phenotype of Children and Adolescents with Selective IgA Deficiency: A Longitudinal Monocentric Study. *J. Clin. Med.* 2022, *11*, 5705”

**DOI:** 10.3390/jcm12237224

**Published:** 2023-11-22

**Authors:** Bianca Laura Cinicola, Giulia Brindisi, Martina Capponi, Alessandra Gori, Lorenzo Loffredo, Giovanna De Castro, Caterina Anania, Alberto Spalice, Cristiana Alessia Guido, Cinzia Milito, Marzia Duse, Isabella Quinti, Federica Pulvirenti, Anna Maria Zicari

**Affiliations:** 1 Department of Maternal Infantile and Urological Sciences, Sapienza University of Rome, 00161 Rome, Italy; giulia.brindisi@uniroma1.it (G.B.);; 2 Department of Molecular Medicine, Sapienza University of Rome, 00161 Rome, Italy; cinzia.milito@uniroma1.it (C.M.);; 3 Department of Translational and Precision Medicine, Sapienza University of Rome, 00161 Rome, Italy; 4 Department of Clinical, Internal Medicine, Anesthesiology and Cardiovascular Sciences, Sapienza University of Rome, 00161 Rome, Italy; 5 Department of Developmental and Social Psychology, Sapienza University of Rome, 00161 Rome, Italy; 6 Department of Internal Medicine and Infectious Diseases, Regional Reference Centre for Primary Immune Deficiencies, Azienda Ospedaliera Universitaria Policlinico Umberto I, 00161 Rome, Italy

We carefully read the correspondence [1] on our manuscript “The Allergic Phenotype of Children and Adolescents with Selective IgA Deficiency: A Longitudinal Monocentric Study” [2]. 

The first issue was about the nomenclature of Inborn Errors of Immunity, which continuously evolves. As stated in the comment, ESID defines the disease of interest as “IgA deficiency” [3]. At the same time, the IUIS classification uses the term “Selective IgA deficiency” in both cases to indicate the lack of IgA (less than 0.07 g/L) with normal other immunoglobulin isotype levels [4]. Some authors make a distinction between “absolute” IgA defect, when the patient’s serum IgA level is less than 0.07 g/L, and “partial”, when the IgA level is at least two standard deviations below that normal for their age [5,6]. Moreover, the term partial has also been associated with the term “selective” by some authors [7,8]. Despite classification efforts, partial and absolute/selective IgA deficiencies cannot be considered exclusive categories, especially in a longitudinal setting, as individuals initially classified as partial IgA deficiency can later fulfil the criteria for absolute IgA deficiency and vice versa. Indeed, in our study, six out of eight patients with partial IgA deficiency seen at enrollment progressively reduced their serum levels to <0.07 g/L. Thus, almost all the patients included in this study fulfilled the criteria of absolute IgA deficiency at least once during the study period.

Secondly, we observed that children with selective IgA deficiency were more likely to have transitory lymphopenia at the initial evaluation than at the end of follow-up. However, we stated that none of the study participants displayed severe lymphopenia. Univocal referral values for the minimum lymphocyte count defining lymphopenia are not available for children, and different studies report similar but not overlapping values, varying accordingly with age. Our study’s median age at the first evaluation was 5.5 years (IQR 3–8 years), and the median age at the last follow-up was 9.0 years (IQR 7–13.5). Then, we calculated the rate of lymphopenia at these two time points using different lymphocyte count ranges based on age, according to Shearer et al. [9]. 

We hope these clarifications might improve the understanding of the results of our study.
